# Case Report: Collision tumors of the sellar region: pituitary adenoma and chordoma coexist in the sellar region

**DOI:** 10.3389/fendo.2025.1605605

**Published:** 2025-06-17

**Authors:** Shuo Gao, Pule Liu, Shude Yang, Kai Liu, Zhiqiang Zhao, Yadong Wang, Qiang Li, Jinqian Dong

**Affiliations:** ^1^ Department of Neurosurgery, The Second Hospital & Clinical Medical School, Lanzhou University, Gansu, China; ^2^ Department of Pathology, The Second Hospital & Clinical Medical School, Lanzhou University, Gansu, China; ^3^ Department of Emergency, Qingyang Xifeng District People’s Hospital, Gansu, China

**Keywords:** collision tumors, chordoma, pituitary adenoma, sellar region, case report

## Abstract

Collision tumors are relatively rare and refer to the coexistence of distinct tumors in the same anatomical region, where they invade each other. Sellar collision tumors are especially uncommon. Recent studies suggest that most cases involve the coexistence of pituitary adenomas and craniopharyngiomas, whereas collision tumors comprising pituitary adenomas and chordomas are exceedingly rare. To the best of our knowledge, this case represents the fourth reported instance of an intrasellar chordoma coexisting with a pituitary adenoma. This case report presents a 60-year-old male patient with a collision tumor consisting of a sellar chordoma and a pituitary adenoma. Enhanced MRI identified a large space-occupying lesion involving the sellar region, suprasellar area, and third ventricle, suspected to be a soft tissue chordoma. Histopathological examination following endoscopic transnasal surgery confirmed the diagnosis of a sellar chordoma coexisting with a pituitary adenoma. This case offers valuable insights into the clinical manifestations and imaging characteristics of sellar chordoma coexisting with pituitary adenoma, contributing to a better understanding of this rare condition. Furthermore, this article reviews the existing literature, serving as a reference for the diagnosis and management of such conditions.

## Introduction

Sellar chordoma is a rare malignant tumor that typically arises from notochordal remnants. Its incidence is low, comprising approximately 1% of all malignant bone tumors and 0.2% of intracranial tumors (1, 2), Most sellar chordomas originate from bones adjacent to or superior to the sella turcica, within the axial skeleton (3). The clinical manifestations of chordoma are primarily associated with tumor growth and invasion, frequently leading to neurological symptoms such as visual impairment, headache, and vomiting (4). Pituitary adenomas are common benign brain tumors, with an incidence of approximately 1 in 1100, typically manifesting as endocrine dysfunction and frequently detected early via imaging studies (5). The concurrent occurrence of sellar chordoma and pituitary adenoma is exceptionally rare in clinical practice (6). To the best of our knowledge, this case represents the fourth reported instance of intrasellar chordoma coexisting with a pituitary adenoma worldwide (6–8). Given the overlapping clinical symptoms of these two tumor types, misdiagnosis or delayed diagnosis is possible. When two histologically distinct tumors coexist in the same anatomical region and invade each other, this phenomenon is termed a collision tumor (9, 10). Despite extensive theoretical discussions on the pathophysiological mechanisms of collision tumors, clinical experience and research data remain limited due to the rarity of sellar collision tumors (9). This study seeks to enhance our understanding of the rare coexistence of sellar chordoma and pituitary adenoma by detailing the clinical presentation, imaging findings, pathological characteristics, and treatment course of this case, thereby providing a reference for future clinical diagnosis and management.

## Case report

A 60-year-old male patient presented to the hospital with a two-month history of headache, dizziness, nausea, vomiting, and blurred vision in the right eye. The patient had no history of chronic diseases and no prior surgical history. He had a smoking history of over 20 years and no known family history of genetic disorders. Physical examination identified a temporal visual field defect in the right eye. Subsequent visual acuity, visual field, and fundus examinations confirmed reduced vision in the right eye compared to the left, with a significant temporal visual field defect ranging from 20° to 30°. Fundus examination revealed no direct lesions. The pituitary and target gland hormones were measured. Endocrine evaluation indicated an elevated thyroid-stimulating hormone (TSH) level (7.341 μIU/ml; reference range: 0.27–4.20 μIU/ml) (**Table 1**), while other hormones were within normal limits. The remainder of the physical examination was unremarkable. Routine blood tests and biochemical analyses, including blood chemistry and electrolyte levels, showed no significant abnormalities on admission.

**Table 1:** Preoperative refers to the endocrine indicator tests conducted upon the patient’s admission, while Postoperative denotes the endocrine indicator tests performed within 24 hours after the patient’s surgery.

Project name	Preoperative	1-day Postoperative	1-month postoperative	Unit	Reference range
T3	1.32	1.51	1.75	nmol/L	1.01-2.96
T4	93.10	130.40	100.30	nmol/L	55.40-161.25
TSH	7.341	3.219	4.020	μIU/ml	0.27-4.20
PRL	11.04	16.64	15.96	ng/ml	2.10-17.70
FSH	10.72	9.74	10.36	mIU/ml	1.40-18.10
LH	6.93	5.79	7.92	mIU/ml	1.50-9.30
ACTH	27.60	22.30	30.30	pg/mL	5.00-46.00(a.m.) 5.00-23.00(p.m.)
GRH	0.42	1.16	0.28	ng/ml	0.00-3.00
IGF-1	96.40	93.70	83.90	ng/ml	31.00-323.00
COR	8.99	10.70	10.90	ug/dl	5.00-25.00(a.m.) 2.50-12.50(p.m.)

T3 represents triiodothyronine, T4 represents thyroxine, TSH represents thyroid stimulating hormone, PRL represents pituitary prolactin, FSH represents follicle stimulating hormone, LH represents luteinizing hormone, ACTH represents adrenocorticotropic hormone, GRH represents growth hormone releasing hormone, IGF-1 represents insulin-like growth factor-1, COR represents cortisol.

**Figure 1:**
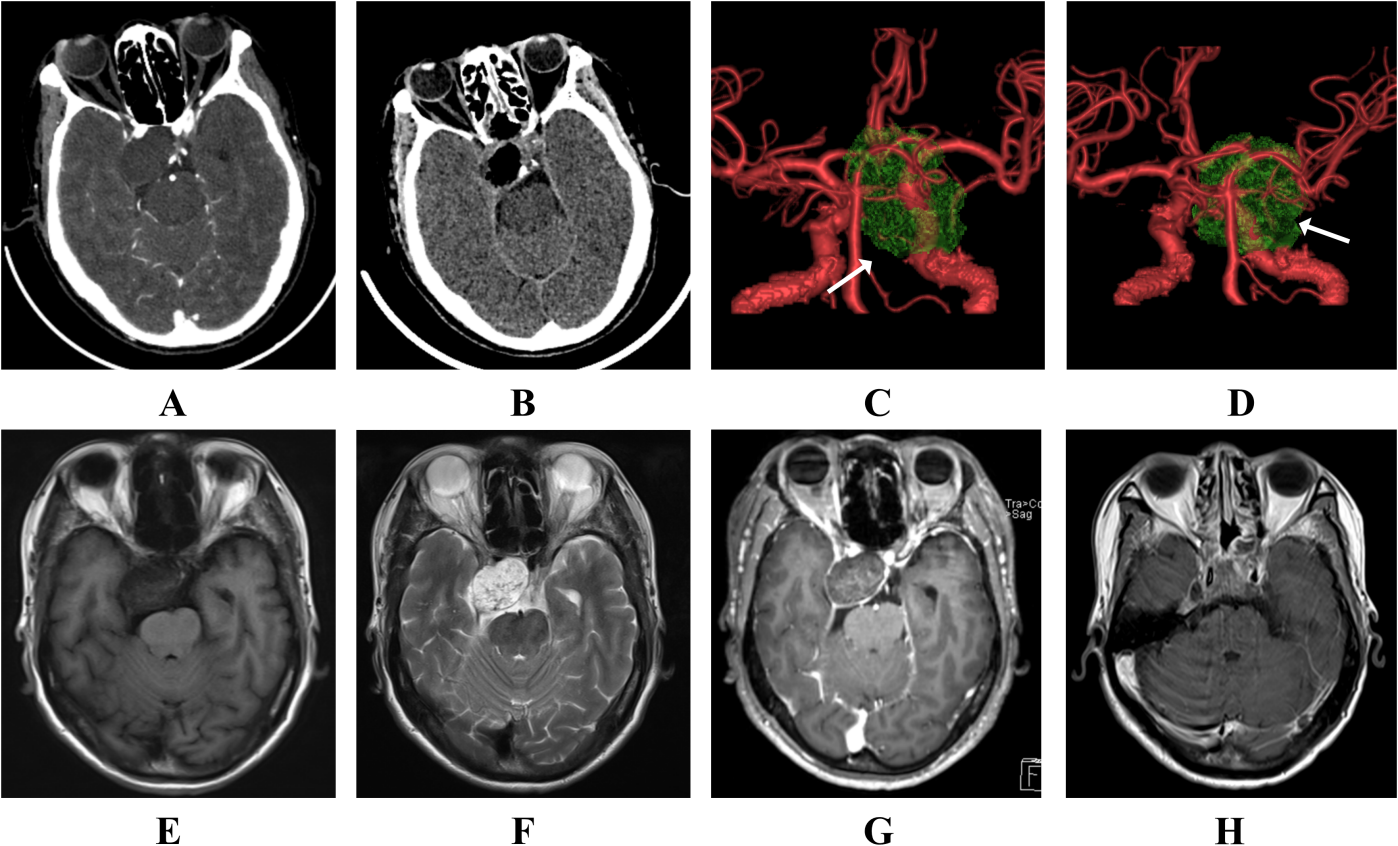
**(A)** shows the patient’s preoperative cranial CT scan, **(B)** shows the postoperative cranial CT scan within 24 hours; **(C, D)** represent the patient’s preoperative cranial CTA images, indicates the tumor invaded the cavernous sinus and encased the right internal carotid artery; **(E, F)** represents the patient’s preoperative MRI T1-weighted imaging (T1WI) and MRI T2-weighted imaging (T2WI), respectively; **(G)** shows the preoperative contrast-enhanced MRI of the patient; **(H)** shows contrast-enhanced MRI at 48 hours postoperatively.

**Figure 2:**
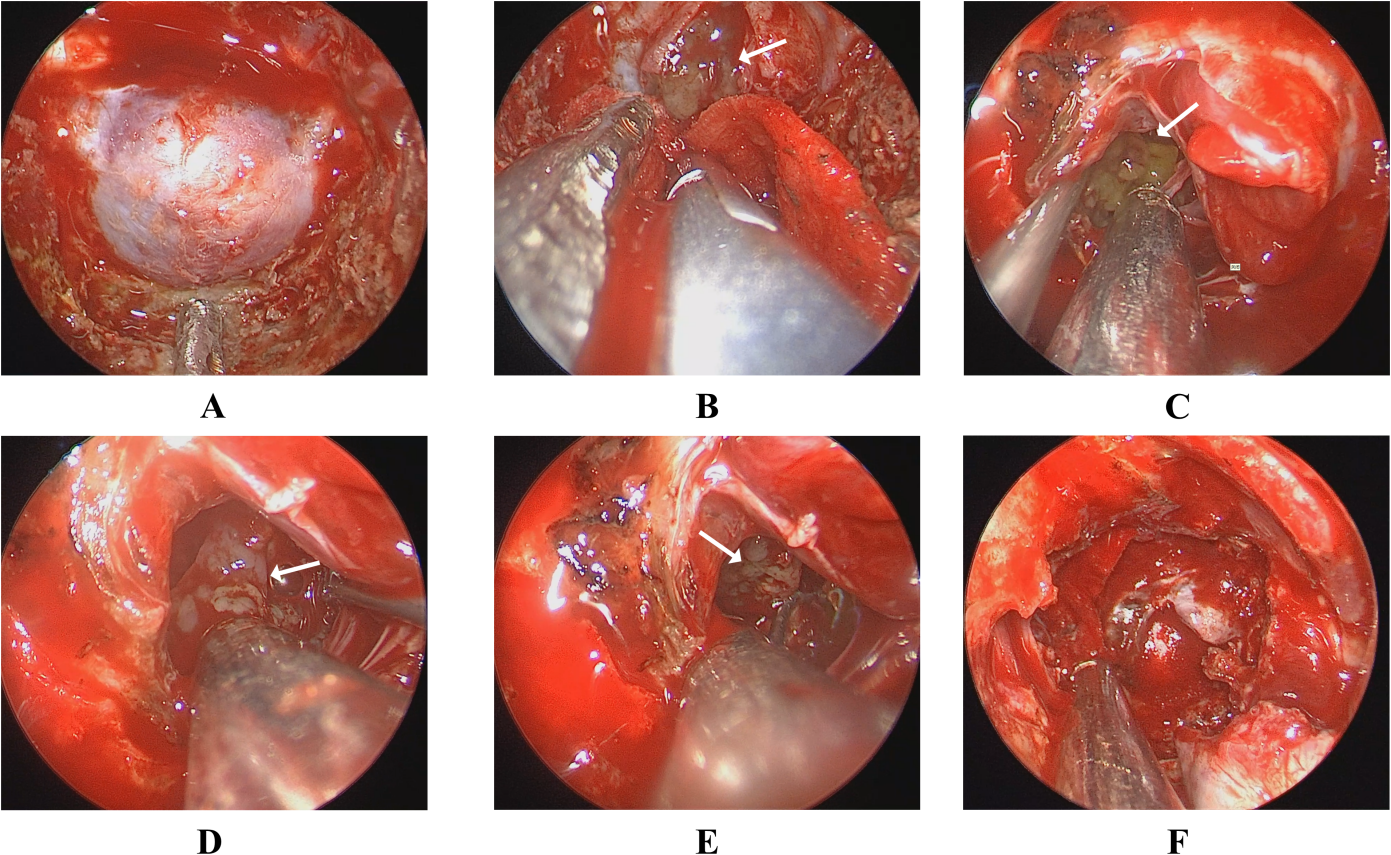
**(A)** shows the intraoperative exposure of the sellar floor; **(B, C)** shows the pale yellow tumor near the pituitary stalk and the third ventricle during surgery; **(D, E)** shows the tumor near the clivus, which appears grayish-white during the operation; **(F)** shows the skull base reconstruction after near-total tumor resection.

**Figure 3:**
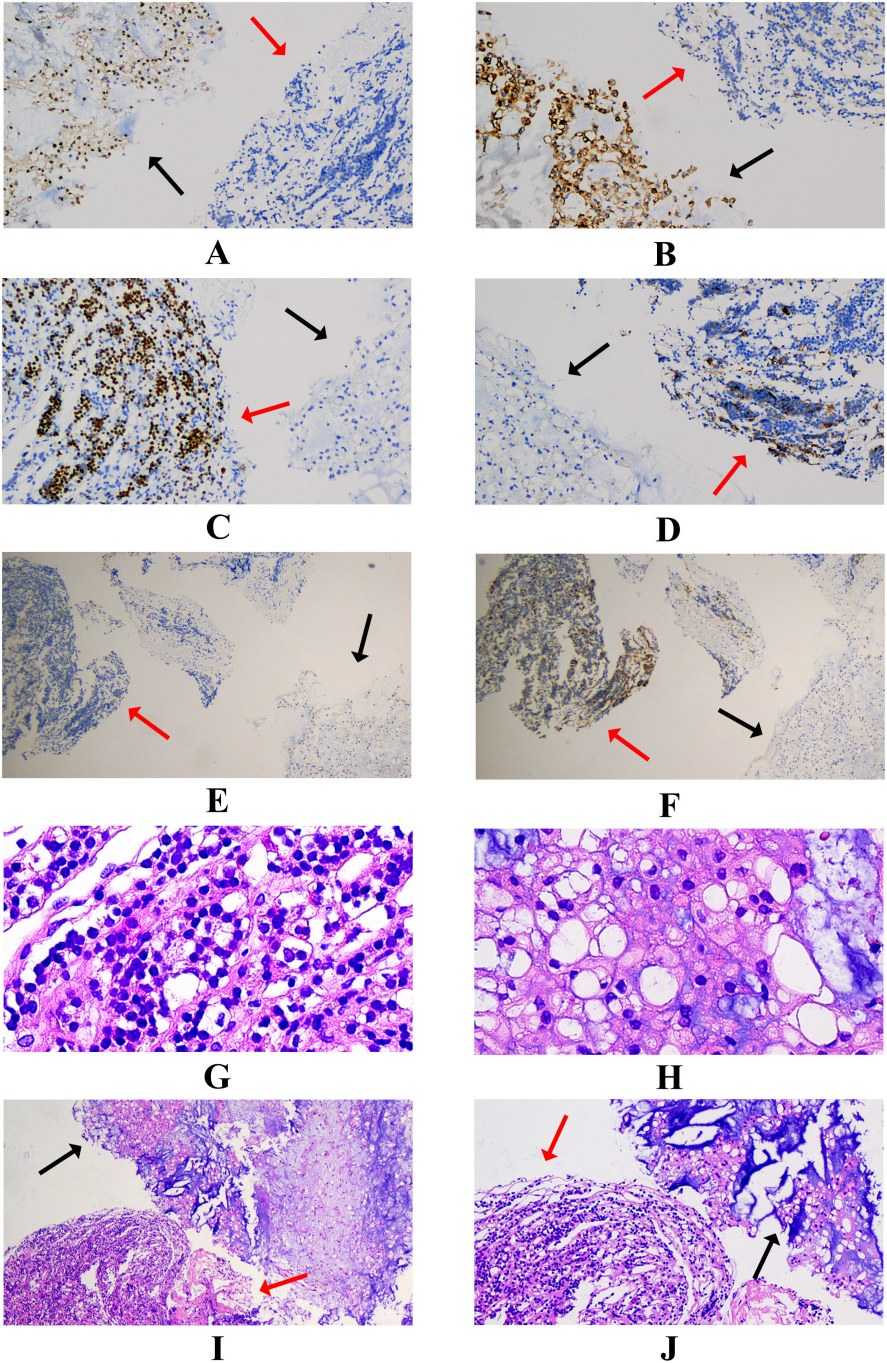
Histopathological examination results. The red arrow indicates pituitary adenoma tissue, and the black arrow indicates chordoma tissue. Panels **(A–F)** show immunohistochemical results of mixed chordoma and pituitary adenoma tissue **(A–D)** ×100, **(E, F)** ×40); **(A)** Brachyury (Chordoma+), **(B)** EMA (Chordoma+), **(C)** PIT-1 (Pituitary neuroendocrine tumor+), **(D)** PRL (Pituitary neuroendocrine tumor+), **(E)** TSH (Pituitary neuroendocrine tumor-), **(F)** GH (Pituitary neuroendocrine tumor+). **(G)** shows pituitary adenoma (×200), **(H)** shows chordoma (×200), **(I)** (×40) and **(J)** (×100) show H&E staining results of mixed chordoma and pituitary adenoma tissue.

The cranial computed tomography (CT) scans reveal bone destruction in the dorsum sellae, indicative of the chordoma (**Figure 1A**). The patient’s CT angiography (CTA) examination revealed a tumor encircling the cavernous segment of the right internal carotid artery (**Figures 1C, D**). Magnetic Resonance Imaging (MRI) showed a roughly oval-shaped mass measuring approximately 23mm×34mm×37mm in the sellar region, suprasellar area, and the third ventricle (**Figure 1G**). On T1-weighted imaging (T1WI), the tumor appeared hypointense with punctate and flaky slightly hyperintense areas (**Figure 1E**), while on T2-weighted imaging (T2WI), it was predominantly hyperintense with local punctate and flaky hypointense areas (**Figure 1F**). The patient was diagnosed with a sellar chordoma, subsequently underwent endoscopic endonasal surgery. However, during the operation, two different types of tumors were found in the sella turcica (**Figure 2**). The tumor tissue near the pituitary structure was soft, pale yellow (**Figures 2B, C**), while the tumor near the clivus position was grayish-white and harder in texture (**Figures 2D, E**). A near-total resection of the tumors was performed. Histopathological results confirmed two different tumors(**Figure 3**): Brachyury (Chordoma+), EMA (Chordoma+), CK8/18 (Chordoma and Pituitary neuroendocrine tumor), CKp (Chordoma and Pituitary neuroendocrine tumor+), PIT-1 (Pituitary neuroendocrine tumor+), GH (+), PRL (Focal +/-), Syn (Pituitary neuroendocrine tumor+), Vimentin (+), GFAP (-), Olig-2 (-), S-100 (-), p53 (Wild type), CK5/6 (Focal +/-), T-PIT (-), SF-1 (-), FSH (-), Ki67 (3%+), ACTH (Focal +/-), TSH (-), LH (-). Therefore, based on histopathology, the final diagnosis in this case was sellar chordoma combined with a PIT-1 lineage pituitary adenoma.

Within 24 hours postoperatively, the patient underwent a CT scan, which revealed a small amount of hematoma and pneumatosis in the surgical area (**Figure 1B**). MRI was conducted 48 hours after surgery, and no notable abnormal enhancement was detected in the surgical site, the tumor was almost entirely resected (**Figure 1H**). Endocrine evaluation within 24 hours postoperatively indicated complete normalization of pituitary hormone levels (**Table 1**). On postoperative day 2, a visual assessment demonstrated improvement in the patient’s right eye vision and visual field compared to preoperative findings. At the 1-month follow-up, the patient had resumed normal daily activities, with endocrine levels remaining within normal limits (**Table 1**). The patient also received radiotherapy at a local hospital and exhibited good tolerance to the treatment. To further investigate the cause of elevated TSH levels, a clinical symptom inquiry focusing on primary hypothyroidism symptoms was conducted, and the patient denied any such symptoms. During the 3-month postoperative follow-up, the patient underwent pituitary and target gland hormone testing at the local hospital, with all results within normal ranges. Currently, the patient has resumed normal occupational activities.

## Discussion

Sellar tumor collision, as a rare clinical phenomenon, refers to the coexistence of a pituitary adenoma with other types of tumors (such as craniopharyngioma or chordoma) within the sellar region (9). These cases present significant diagnostic challenges because their clinical presentation and imaging features are often too similar to those of isolated sellar tumors (10, 11), requiring multidisciplinary collaboration and high vigilance for accurate diagnosis and effective treatment. In this case, the patient had both a pituitary adenoma and a chordoma, which fully illustrates the complexity of this rare tumor collision phenomenon and warrants further in-depth exploration.

Pituitary adenomas are typically caused by hyperplasia of glandular cells within the pituitary gland and often manifest as endocrine dysfunction, with clinical symptoms including hyperprolactinemia, adrenal insufficiency, hypothyroidism, and hypopituitarism (5). In the present case, preoperative endocrine testing revealed only a borderline elevation in TSH levels, while other hormone levels were within normal limits. As a result, the potential diagnosis of a pituitary adenoma was not sufficiently considered preoperatively, and imaging findings led to a misdiagnosis of chordoma. This misdiagnosis may have been due to radiologic similarities and a lack of awareness of atypical presentations of pituitary adenomas. Additionally, non-functioning pituitary adenomas may not present with overt endocrine abnormalities but can cause compressive symptoms such as headache and visual disturbances as the tumor enlarges (12). The most common visual impairment is bitemporal visual field defects, with the upper and peripheral fields most prominently affected (5).

Chordoma is a rare tumor arising from notochordal remnants (1), Although intrasellar chordoma is exceptionally rare, several studies have documented cases where it mimicked a pituitary adenoma (13, 14). Furthermore, three cases of chordomas coexisting with pituitary adenomas have been previously reported (6–8). The first reported case described a parasellar chordoma coexisting with a non-functioning pituitary adenoma, the second case involved a chordoma coexisting with a prolactinoma, whereas the third case featured a cystic chordoma alongside a non-functioning pituitary adenoma. Thus, this current case may constitute the fourth reported instance of a chordoma coexisting with a pituitary adenoma in the sellar region. Owing to its distinct anatomical location, intrasellar chordoma frequently induces compressive symptoms. Common symptoms include headache, blurred vision, vomiting, and cranial nerve palsy (4). It is noteworthy that chordomas and pituitary adenomas share overlapping clinical manifestations to some extent, necessitating a high index of clinical suspicion for their potential coexistence. In this case, the patient presented only with neurological deficits caused by compression from the sellar mass, without any symptoms of pituitary dysfunction, which increased the diagnostic challenge and may have hindered timely and accurate judgment by the neurosurgical team.

Pituitary adenomas and chordomas have distinct imaging features. Pituitary adenomas typically present as soft tissue masses in the sellar region, exhibiting low signal intensity on T2WI in approximately two-thirds of cases (15). In contrast, chordomas generally display low to intermediate signal intensity on T1WI and high signal intensity on T2WI (16, 17). Although pituitary adenomas and chordomas have distinct imaging features, their close anatomical proximity and potential interactions can lead to overlapping imaging findings, heightening the risk of misdiagnosis or overlooked diagnosis. Imaging evaluation must incorporate clinical symptoms, endocrine assessment, and the possibility of tumor collision. The accuracy of imaging-based diagnosis depends on tumor size, location, and complexity, particularly when clinical symptoms are ambiguous. Advances such as functional MRI and PET-CT have enhanced detection capabilities; however, clinical judgment remains indispensable.

Treatment options for sellar collision tumors are relatively limited, the primary treatment strategy remains surgical resection, guided by tumor size and location (5, 6). Previous reports on sellar collision tumors have documented two cases in which inaccurate diagnoses led to a second surgical intervention. Due to the rarity of sellar collision tumors and the failure to achieve an accurate diagnosis based on pathological and imaging characteristics, these patients underwent a second surgery, resulting in additional suffering (10, 18). The management of pituitary adenomas is primarily dictated by tumor functionality, size, and the patient’s overall health status (5). Functional adenomas, such as prolactinomas and ACTH-secreting tumors, often respond well to pharmacological treatment (19). whereas nonfunctional or large tumors typically require surgical intervention (5, 20). Conversely, the management of chordomas is more complex, often necessitating intricate surgical procedures (21). In this case, the tumor infiltrated the clivus and encased the right internal carotid artery. Considering the tumor’s growth pattern and anatomical location, meticulous surgical techniques were required to mitigate the risk of injury to adjacent structures. Following surgical resection, adjuvant radiotherapy or chemotherapy may be necessary for some chordoma patients to reduce the likelihood of recurrence (1, 22). Given the high recurrence rate of chordomas, extended postoperative monitoring is imperative.

Pituitary adenomas generally have a favorable prognosis, as early intervention facilitates endocrine recovery and reduces recurrence rates (5). However, chordomas exhibit a propensity for recurrence, particularly when complete resection is unachievable (22). Due to the absence of a standardized treatment protocol for sellar collision tumors, individualized management and multidisciplinary collaboration—encompassing neurosurgery, endocrinology, radiology, and oncology—are essential for optimizing patient outcomes.

## Conclusions

This case underscores the significance of considering tumor collision in the diagnosis of sellar tumors, particularly when faced with complex symptoms or imaging findings. The coexistence of pituitary adenomas and chordomas is exceptionally rare. However, with advancements in imaging technology and the accumulation of clinical expertise, the accuracy of diagnosis and treatment has progressively improved. For such patients, a comprehensive approach incorporating clinical symptoms, imaging studies, pathological results, and multidisciplinary collaboration offers more precise and personalized treatment plans, thereby improving treatment outcomes and prognosis. In the future, as additional cases of sellar collision tumors are reported, our understanding of this phenomenon will advance, providing a more robust theoretical foundation for the early detection and treatment of related diseases.

## Data Availability

The original contributions presented in the study are included in the article/supplementary material. Further inquiries can be directed to the corresponding author.
